# Integrated Analysis of Metabolome and Volatile Profiles of Germinated Brown Rice from the *Japonica* and *Indica* Subspecies

**DOI:** 10.3390/foods10102448

**Published:** 2021-10-14

**Authors:** Chenguang Zhou, Yaojie Zhou, Yuqian Hu, Bin Li, Roujia Zhang, Kaiyi Zheng, Jie Liu, Jing Wang, Min Zuo, Siyao Liu

**Affiliations:** 1 School of Food and Biological Engineering, Jiangsu University, Zhenjiang 212013, China; zhouchenguang@ujs.edu.cn (C.Z.); ZhouYaojie2021@gmail.com (Y.Z.); hu13636001423@163.com (Y.H.); lb13753676201@163.com (B.L.); 1000005191@ujs.edu.cn (R.Z.); kaiyizhengjsu@126.com (K.Z.); 2 China-Canada Joint Lab of Food Nutrition and Health (Beijing), Beijing Technology and Business University, Beijing 100048, China; liu_jie@btbu.edu.cn (J.L.); wangjing@th.btbu.edu.cn (J.W.); 3 National Engineering Laboratory for Agri-Product Quality Traceability, Beijing Technology and Business University, Beijing 100048, China; zuomin1234@163.com; 4 School of Pharmacy, Jiangsu University, Zhenjiang 212013, China

**Keywords:** brown rice, metabolomics, volatile, GC-MS, HS-SPME

## Abstract

In the present study, germinated brown rice (GBR) from three *Japonica* and three *Indica rice* cultivars were subjected to metabolomics analysis and volatile profiling. The statistical assessment and pathway analysis of the metabolomics data demonstrated that in spite of significant metabolic changes in response to the germination treatment, the *Japonica rice* cultivars consistently expressed higher levels of several health-promoting compounds, such as essential amino acids and γ-aminobutyric acid (GABA), than the *Indica* cultivars. No clear discriminations of the volatile profiles were observed in light of the subspecies, and the concentrations of the volatile organic compounds (VOCs), including alkenes, aldehydes, furans, ketones, and alcohols, all exhibited significant reductions ranging from 26.8% to 64.1% after the germination. The results suggest that the *Japonica* cultivars might be desirable as the raw materials for generating and selecting GBR food products for health-conscious consumers.

## 1. Introduction

Rice (*Oryza sativa*) is one of the most important cereal grains and is consumed as a staple food for a large part of the world’s population. In the course of refined-rice processing, the outer bran and embryo of intact rice grains are removed to generate white rice, which is mainly composed of starchy endosperm [1]. In comparison, brown rice (BR) is an unpolished whole-grain cereal which consists of an outer bran layer, an embryo, and an endosperm. Brown rice is well-known to be rich in numerous bioactive compounds, including dietary fiber, amino acids, phytosterols, phenolic compound, and γ-aminobutyric acid (GABA) [2]. Researchers reported that a higher consumption of white rice is associated with an increased risk of type 2 diabetes, and the intake of whole-grain brown rice instead may be one of the strategies to prevent this [3,4].

Although its consumption has an undeniable health-promoting effect, brown rice is not considered suitable as a staple food owing to its hard texture and not easy-to-cook characteristic [5]. Germination is regarded as a domestic and economic strategy for reducing the difficulty of cooking as well as for improving the palatability of brown rice [6]. Moreover, targeted analyses have revealed that the germination process notably enhanced the concentration of nutrients and health-prompting compounds in brown rice. Ti et al. [7] reported significant increases of free and bound phenolic compounds in germinated brown rice (GBR) at different germination stages. Ng et al. [8] revealed the remarkable rise in the amounts of tocopherols, tocotrienols, and γ-oryzanol in GBR after gemination treatment.

Compared with the above-mentioned targeted analyses of constituents, metabolomics nowadays has been applied to reveal a more comprehensive metabolite mapping of cereal grains. For example, non-targeted metabolite profiling was employed to reveal the metabolic changes after seed germination of the *Indica* cultivar DHX [9]. Na Jom et al. [10] compared the metabolic differences of four Thai colored *Indica* GRB with the use of the metabolite profiling approach. Currently, a limited amount of information is available only for *Indica* GBR regarding the global metabolic characterization, while no metabolomics-based investigation of *Japonica* GBR has ever been reported.

Rice cultivars are mainly grouped into *Japonica* subspecies and *Indica* subspecies based on the geographical distribution, plant architecture, and physiological features [11]. Usually, *Japonica rice* grains are short, roundish, and sticky, and are often consumed in North China. By contrast, *Indica rice* kernels are slender and more popular in South China. Accordingly, the overall metabolite profiles of the GBR generated from these two subspecies are expected be to distinct. However, limited information is available regarding the metabolic differentiation between the *Japonica* GBR and the *Indica* GBR. Additionally, it also remains unclear whether the volatile profiles of the *Japonica* GBR and the *Indica* GBR are different.

In the present study, a gas chromatography–mass spectrometry (GC-MS)-based metabolomics approach was employed to comprehensively characterize the metabolite profiles of GBR from three *Indica* and three *Japonica rice* cultivars. In addition, the head-space solid-phase microextraction GC-MS (HS-SPME-GC-MS) method combined with chemometric analysis were utilized to explore the volatile profiles of the GBR resulting from the *Japonica* and the *Indica* subspecies. The objectives of this study were: (i) to investigate the changes in metabolite profiles and volatiles in rice seeds upon germination treatment, and (ii) to compare the global metabolic and volatile differences between the *Japonica* and the *Indica* GBR. The elaborated data are expected to increase the knowledge in generating and selecting GBR food products for health-conscious consumers.

## 2. Materials and Methods

### 2.1. Chemicals and Reagents

The internal standard 4-chloro-l-phenylalanine and 1-octanol were supplied by Macklin Inc. (Shanghai, China). The alkane standard mixture (C7 to C40 straight-chain alkanes) was obtained from Sigma-Aldrich (Steinheim, Germany). Other chemicals, including chloroform, methoxyamine hydrochloride, pyridine, and *N*-Methyl-*N*-trimethylsilyl trifluoroacetamide (MSTFA) were purchased from Sinopharm Chemical Reagent (Shanghai, China).

### 2.2. Sample Materials and Germination Process of Brown Rice 

In the present study, the brown rice (BR) from six non-fragrant cultivars widely cultivated in China, i.e., Ningjing 43 (*Japonica rice*, hereafter NJ), Longjing 25 (*Japonica rice*, hereafter LJ), Tonghe 899 (*Japonica rice*, hereafter TH), Zhongzao 39 (*Indica rice*, hereafter ZZ), Zhongjiazao 17 (*Indica rice*, hereafter ZJZ), and Luoyou 8 (*Indica rice*, hereafter LY) were used. For germination treatment, fifty grams of brown rice seeds from each cultivar were washed by distilled water and soaked in 10 mL/L sodium hypochlorite for 30 min. Then, all the surface-sterilized rice seeds were drained and soaked in distilled water at 30 °C for 12 h. The soaking water was changed every 4 h. The soaked rice seeds were subsequently placed on three pieces of wet filter papers in plastic germination trays and incubated in a growth chamber (Life Apparatus Company, Ningbo, China). The germination process was carried out in the growth chamber at 25 °C under a 16 h light/8 h dark photoperiod for 72 h. After the germination process, the collected GBR samples were flash-frozen in liquid nitrogen and ground into flour using a rice mill. Finally, the rice flour was lyophilized and stored at −80 °C for further analysis.

### 2.3. Sample Preparation and Metabolomics Analysis

Extraction and derivatization of metabolites were performed according to the method previously described in [12], with slight modifications. Briefly, 50 mg of freeze-dried samples were weighed into a centrifuge tube and 1 mL of precooled 100% methanol was added. Twenty microliters of 4-chloro-L-phenylalanine (1.5 mg/mL in water) were added as an internal standard (IS). After vortexing and homogenization, the mixture was ultrasonically extracted for 30 min and then centrifuged at 11,000× *g* for 10 min. The supernatant was pipetted into another centrifuge tube, then 300 µL of chloroform and 600 µL of deionized water were added. After centrifugation at 2200× *g* for 15 min, the supernatant was collected and dried using a vacuum concentrator without heating. The methoxime-derivatization process was performed by adding 40 µL of methoxyamine hydrochloride (20 mg/mL in pyridine), followed by shaking at 37 °C for 2 h using a thermomixer. Then, 70 µL of N-Methyl-*N*-trimethylsilyl trifluoroacetamide (MSTFA) was added and the mixture was incubated at 37 °C for 30 min. Afterward, the prepared samples were transferred to glass vials. For each sample, six biological replicates were prepared.

Meanwhile, a quality control (QC) sample was prepared by pooling the same amount (10 g) of all the investigated samples. The QC sample was regularly inserted into the analytical sequence of the actual samples to monitor the reproducibility of the analytical method and check the potential contaminations in the laboratory.

Gas chromatograph–mass spectrometer (GC-MS) analyses were performed using a GCMS-TQ8040 (Shimadzu, Kyoto, Japan) coupled with a DB-5 fused silica capillary column (30 m × 0.25 mm × 0.25 μm; Agilent Technologies, Santa Clara CA, USA), and the instrument parameters were set, as previously described [12].

### 2.4. Metabolomics Data Processing

Raw data files (“qgd” format) generated from the GCMS-TQ8040 were converted to “abf” format with the Abf converter (http://www.reifycs.com/AbfConverter/, accessed date 15 May 2021). Baseline filtering, deconvolution, peak alignment, peak height normalization, and peak identification were performed using MS-DIAL software (http://prime.psc.riken.jp/compms/msdial/main.html, accessed on 15 May 2021) equipped with a Fiehn BinBase DB Library [13]. The average peak width of 20 scans and the minimum peak height of 20,000 amplitudes were adopted for peak detection. The metabolite features that showed relative standard deviations (RSDs) < 30% in the QC samples and were detected in over 80% of the samples were included for further analysis. For the deconvolution parameters, the sigma window value was set to 0.5 and the EI spectra cut-off was set to 5000 amplitudes. In the identification tab, the alkanes (C7-C40)-based retention index (RI) type was selected. The RI tolerance was 20, the cut-off for the EI similarity library tolerance was 70%, the *m*/*z* tolerance was 0.5 Da, and the identification score cut-off was 70%. In the alignment parameter setting tab, the retention time tolerance was set to 0.075 min. Other parameters in MS-DIAL were set to default.

### 2.5. Volatile Analysis by HS-SPME-GC-MS

To simulate the process of cooking rice that is normally performed for consumption, 5 g of the brown rice (BR) and the germinated brown rice (GBR) from each of the rice cultivars, and 7.5 g of distilled water for every 5 g of rice, were combined in 20 mL headspace glass vials. The glass vials were loosely sealed with 18 mm magnetic screw craps fitted with a polytetrafluoroethylene (PTFE) silicone septum and were kept boiling for 30 min in a rice cooker until no white spots were leaching out. After the addition of 50 μL of the internal standard (1-octanol, 0.05 mg/mL in water) to the cooked rice samples, the automatic SPME sampler (AOC-6000 Plus autosampler, Shimadzu) equipped with a divinylbenzene (DVB)/carboxen (CAR)/polydimethylsiloxane (PDMS) 50/30 μm fiber (10 mm, Supelco, PA, USA) was employed to extract the headspace volatiles of cooked rice. After the equilibration of cooked rice samples at 60 °C for 15 min, the pre-conditioned (200 °C for 10 min) SPME fiber was inserted into the glass vial. The samples were agitated at 300 rpm and the extraction lasted for 30 min. Subsequently, the fiber was thermally desorbed in the GC injector port at 250 °C for 5 min. Instrumental analyses were carried out by a GCMS-TQ8040 (Shimadzu, Kyoto, Japan) coupled with a DB-WAX fused silica capillary column (30 m × 0.25 mm × 0.25 μm; Agilent Technologies, Santa Clara, CA, USA) under conditions previously described [14]. The volatile compounds were tentatively identified by comparing the mass spectra with the NIST 17 mass spectral library and the Kovats’ retention index (RI) (calculated from C7 to C40 alkanes) with the NIST Chemistry WebBook database (https://webbook.nist.gov/chemistry/name-ser/, accessed on 20 May 2021). The concentrations of the volatile compounds were expressed as 1-octanol (internal standard) equivalents, and all the response factors were regarded as 1.0.

### 2.6. Data Analysis

For the multivariate analysis, the metabolomics and volatile data matrices were imported into MetaboAnalyst 5.0 [15] for principal component analysis (PCA) and orthogonal partial least square discriminant analysis (OPLS-DA) after log transformation and Pareto scaling pretreatment. The permutation test numbers of the OPLS-DA model were set to 1000.

Statistically significant differences among the contents of metabolites and volatile compounds were determined by Student’s *t*-test and one-way analysis of variance (ANOVA) with Tukey’s post hoc test using the software XLSTAT (version 19.5, Addinsoft, Paris, France) when the features showed a normal distribution and homoscedasticity; otherwise, nonparametric statistical analyses with the Mann–Whitney test and Kruskal–Wallis test were conducted. 

## 3. Results 

In the present study, the rice samples from three *Japonica rice* cultivars (NJ, LJ, and TH) and three *Indica* cultivars (ZZ, ZJZ, and LY) were all subjected to the GC-MS-based nontargeted metabolomics analysis. The obtained raw data were further subjected to the MS-DIAL software for data pretreatment and peak identification. In total, 89 metabolites, including lipids, sugars, sugar alcohols, amino acids, organic acids, amino acids, and amines were tentatively identified according to the mass spectra and the retention index (Supplementary Materials Table S1, Figures S1 and S2). In addition, the QC samples clustered closely in the center of the PCA score plot (Figure S3), indicating the good stability and reproducibility of the employed methodology.

### 3.1. Metabolic Changes in BR upon the Germination Process

**Figure 1:**
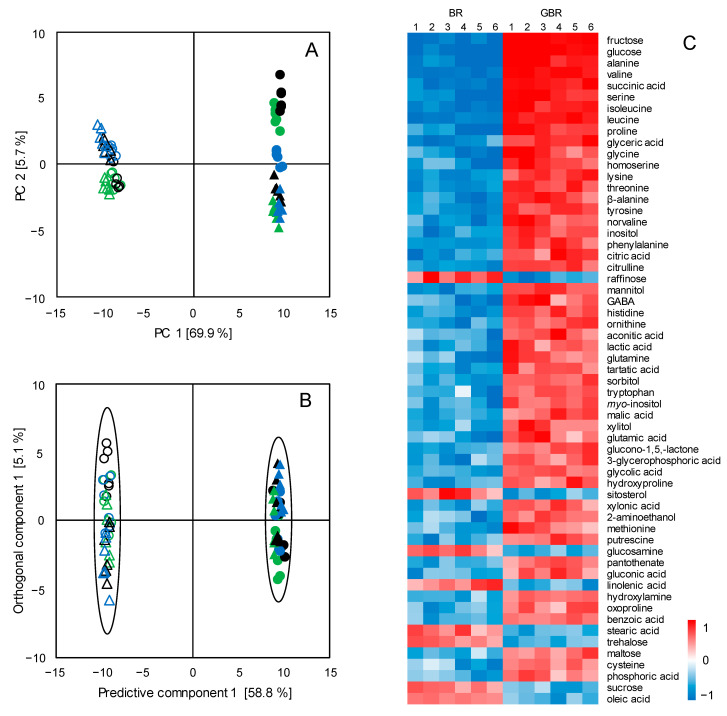
(**A**) PCA score plot of the metabolomics data of brown rice (BR, open) and germinated brown rice (GBR, solid). (**B**) OPLS-DA score plot of the metabolomics data of BR (open) and GBR (solid); the boundaries of the clusters correspond to the 95% Hotelling’s T2 ellipses. (**C**) Heat map of the metabolites contributing to the metabolic discrimination between brown rice (BR) and germinated brown rice (GBR). Lanes 1–3: *Japonica rice* cultivars NJ, LJ, and TH; lanes 4–6: *Indica rice* cultivars ZZ, ZJZ, and LY.

To have an overview of the metabolite profiles of all the BR and GBR samples investigated in the present study, a principal component analysis (PCA) was conducted. As shown in Figure 1A, the PCA score plot exhibited a distinct separation between BR and GBR along PC1, which explained 69.9% of the total variance. On the contrary, no clear discriminations were observed within each clustering. These results indicate significant metabolic changes in BR after the germination process, independent from the subspecies and cultivars of the investigated rice samples. 

To identify the metabolites contributing to the metabolic differentiation between the BR and the GBR, a supervised orthogonal partial least square discriminant analysis (OPLS-DA) was performed. Comparable discrimination between the BR and the GBR was observed along the predictive component (Figure 1B). The cross-validation parameters and the results of the permutation test demonstrated the robustness of the constructed OPLS-DA model without overfitting (Figure S4). Subsequently, the discriminating metabolites were selected on the basis of the VIP values (>1) and the FDR *p*-values (<0.05); a heat map was employed to visualize the variance of these discriminating metabolites (in order of the VIP values).

Figure 1C depicts the detected metabolites exhibiting changed levels in response to the germination treatment. A broad spectrum of metabolites, including sugars (e.g., fructose and glucose), amino acids (e.g., alanine, valine, and serine), organic acids (e.g., succinic acid and citric acid), and lipids (e.g., sitosterol and stearic acid), all presented significantly increased levels after the germination process for both the *Japonica* (Figure 1C: lanes 1, 2, and 3) and the *Indica* (Figure 1C: lanes 4, 5, and 6) rice cultivars. In contrast, only a few metabolites, i.e., raffinose, sucrose, trehalose, glucosamine, sitosterol, stearic acid, oleic acid, and linolenic acid, showed lower levels in the GRB than in the BR. These results suggest that the germination process induced similar metabolic changes in all the investigated BR samples, regardless of the subspecies of the rice samples.

### 3.2. Metabolic Differentiations between Japonica and Indica Rice Subspecies 

**Figure 2:**
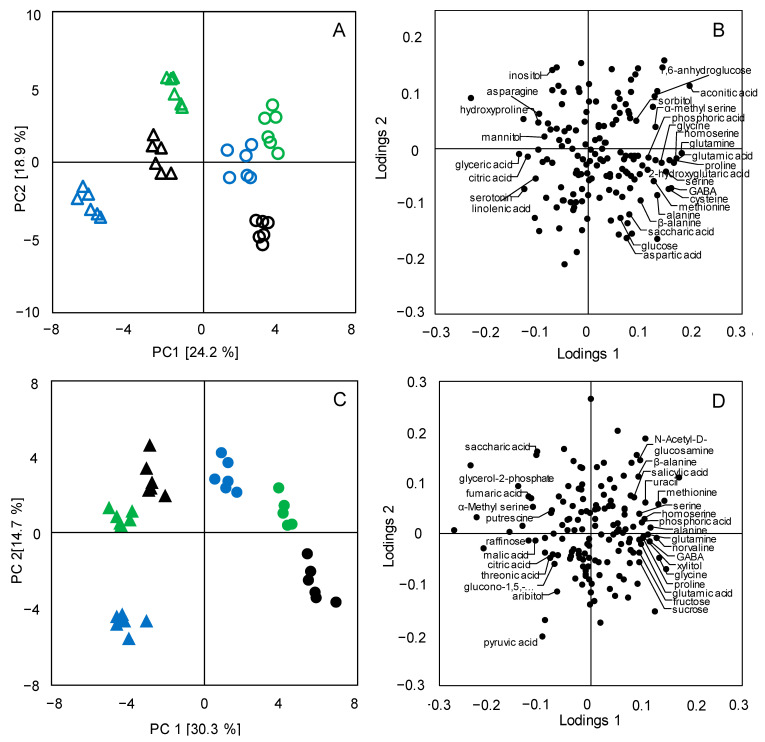
PCA score plots (**A**,**C**) of the metabolomics data of brown rice (open) and germinated brown rice (solid). The samples are from the *Indica* cultivars (triangle) ZZ (green), ZJZ (black), and LY (blue), as well as from the *Japonica* cultivars (open circle) NJ (green), LJ (black), and TH (blue); PCA loadings plots (**B**,**D**) were generated from the corresponding PCA score plots.

Because of the drastic metabolic changes during the germination process, the differentiation of the metabolite profiles according to rice subspecies could not be clearly reflected (Figure 1A). Therefore, individual PCA analyses were conducted for both the BR samples and the GBR samples. As shown in Figure 2A, a clear separation between the *Japonica* BR and the *Indica* BR was observed along PC 1. For the PCA score plot of the metabolomics data of the GBR (Figure 2C), a similar metabolic separation between the *Japonica* GBR and the *Indica* GBR was also seen along PC 1. These results suggest a remarkable metabolic differentiation between the *Japonica* brown rice and the *Indica* brown rice, and such differentiations were consistently expressed after the germination process in GBR.

To identify the differential metabolites contributing to the observed discrimination between the two groups of rice subspecies, the corresponding loadings were plotted (Figure 2B,D). It could be seen that a number of discriminating metabolites highlighted in the loading plot for the BR samples (Figure 2B), e.g., serine, homoserine, glutamic acid, alanine, citric acid, saccharic acid, and GABA, were also observed in the loading plot for the GBR samples (Figure 2D), indicating that the metabolic variations between the *Japonica* BR and the *Indica* BR remained consistent even after the germination process. In addition, some metabolites presented in the loading plot for GBR samples (Figure 2D) were not observed in that for BR samples (Figure 2B), e.g., sucrose, fructose, raffinose, fumaric acid, malic acid, and threonic acid. This result suggests that the metabolic differentiation between *Japonica* and *Indica* GBR was further promoted via the germination process in accordance with the results from the PCA analysis, which showed a higher proportion of the explained total variance along PC 1 for GBR (30.3%, Figure 2C) as compared with that for BR (24.2%, Figure 2A).

### 3.3. Metabolic Pathway Analysis 

**Figure 3:**
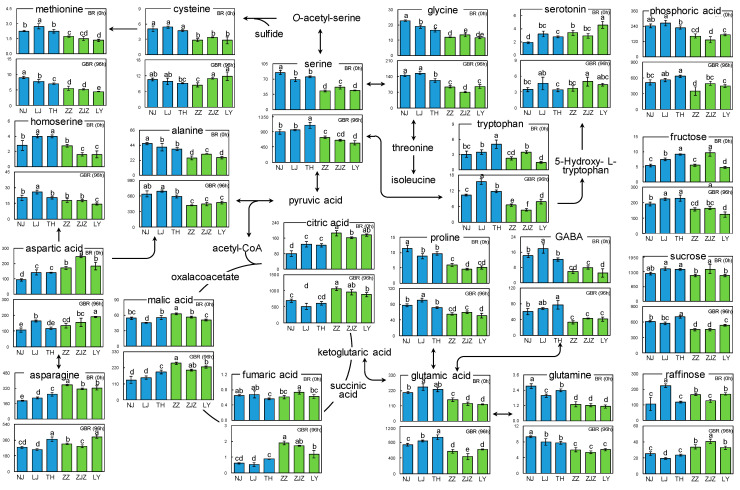
Simplified biosynthetic pathways of the metabolites responsible for the metabolic differentiations between the *Japonica* (blue) and *Indica* (green) cultivars of brown rice (upper cell) and germinated brown rice (lower cells). For each metabolite, different letters indicate statistically significant differences (*p* < 0.05).

To illustrate the subspecies-dependent differentiations in the metabolome, the discriminating metabolites were mapped in a simplified pathway according to the Plant Metabolic Net (PMN) and the Kyoto Encyclopedia of Genes and Genomes (KEGG). As shown in Figure 3, the *Japonica* BR generally presented higher levels of the nitrogen-containing constituents than the *Indica* BR (comparisons in the upper cells for each metabolite). For example, despite the variations in the amounts of serine among the BR samples NJ, LJ, and TH, the *Japonica* BR all exhibited statistically significant higher levels than the *Indica* BR samples ZZ, ZJZ, and LY. Similar results were also observed in other nitrogen-containing metabolites, i.e., glycine, alanine, methionine, proline, glutamic acid, glutamine, and GABA. Moreover, such subspecies-dependent metabolic distinctions were consistently expressed even after the germination process for GBR samples, and most of these nitrogen-containing metabolites still exhibited higher levels in the *Japonica* GBR than in the *Indica* GBR (lower cells for each metabolite).

By contrast, the contents of the energy-storing carbohydrates, e.g., fructose, sucrose, and raffinose, were cultivar-dependent and showed no differentiations with regard to the subspecies (Figure 3). However, during the germination process, all the *Japonica* GBR showed statistically significant higher levels of fructose and sucrose, and lower levels of raffinose, when compared with the *Indica* GBR. Such similar results of the subspecies-dependent metabolic variations in the GBR were also observed in the organic acids such as citric acid, malic acid, and fumaric acid. These results indicate that during the seed germination process, the *Japonica* BR and the *Indica* BR displayed slightly different capacities in energy production and remobilization [16].

### 3.4. Changes in Volatile Profiles Induced by Germination

The volatile organic compounds (VOCs) generated by the BR and the GBR were extracted and determined via HS-SPME-GC-MS. According to the mass spectra data from the NIST 17 library and the Kovats retention index (RI), a total of 80 volatile compounds were identified, including 8 alkanes, 4 alkenes, 22 aldehydes, 9 furans, 12 ketones, 10 alcohols, 5 fatty acid methyl esters, and 10 compounds grouped as others (Table S2), and the concentrations of the identified VOCs in the BR and the GBR were expressed as 1-octanol (internal standard) equivalents. Similar compositions of the volatile profiles were also reported for the BR from other non-fragrant rice cultivars [14,17].

**Figure 4:**
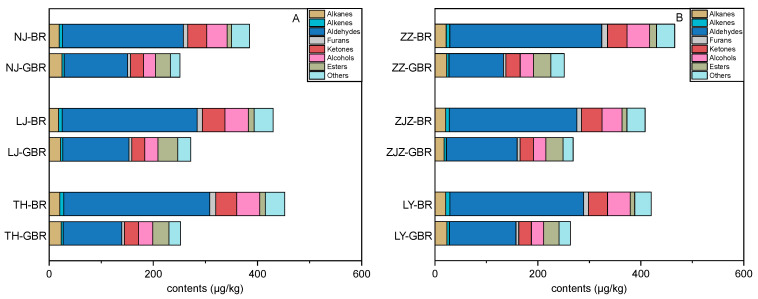
Stacked bar chart of the different chemical classes of the volatile organic compounds detected in brown rice (BR) and germinated brown rice (GBR) from the *Japonica* (**A**) and *Indica* (**B**) cultivars.

Among the eight chemical classes of the detected VOCs, aldehydes were the most abundant volatiles ranging from 239.1 μg/kg to 280.1 μg/kg in the *Japonica* BR (Figure 4A), in the same order of magnitude as the previously reported data [17,18]. Compared with the contents of the aldehydes in the *Japonica* BR, the remarkable reductions of up to 60.1% were observed for the *Japonica* GBR after the germination process. Such drastic decreases in the contents of the VOCs were also observed for the other chemical classes such as alkanes, furans, ketones, and alcohols, with the exception of alkanes, which showed slightly increased levels in all the investigated *Japonica* GBR upon germination treatment (Figure 4A). For the *Indica rice* ZZ, ZJZ, and LY, comparable reduction degrees in the amounts of alkenes (34.1–40.5%), aldehydes (44.5–64.1%), furans (37.7–58.1%), ketones (26.8–34.6%), and alcohols (38.4–45.4%) were observed in the GBR, respectively, when compared with those in the corresponding *Indica* BR samples (Figure 4B). These results reveal that the germination process significantly decreases the VOC levels in the GBR, independent of the rice subspecies and cultivars, thus leading to the negative impact on the perception of the cooked whole-grain rice.

**Figure 5:**
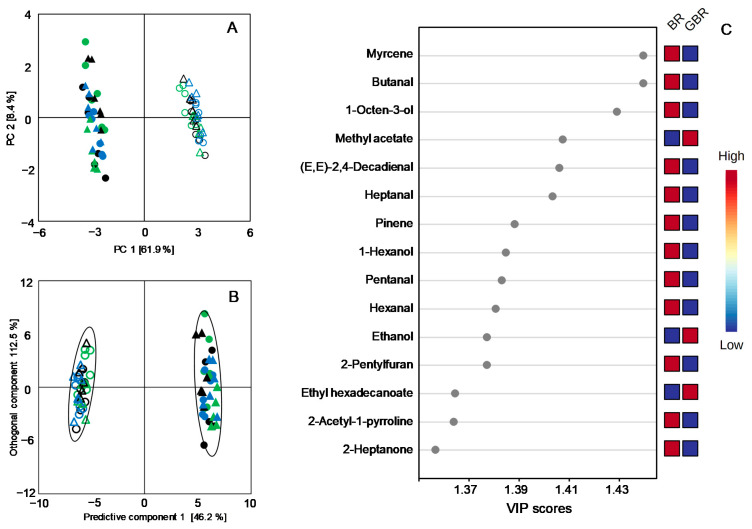
(**A**) PCA score plot of the volatile profiling data of BR (open) and GBR (solid). (**B**) OPLS-DA score plot of the volatile profiling data of brown rice (open) and germinated brown rice (solid); the boundaries of the clusters correspond to the 95% Hotelling’s T2 ellipses. (**C**) Heat map of the selected discriminating VOCs contributing to the separations of the volatile profiles of brown rice and germinated brown rice.

To examine the aroma compounds whose contents were most significantly changed upon germination, an unsupervised PCA and a supervised OPLS-DA were conducted. The VIP score filtering was subsequently utilized to identify the volatile compounds contributing to the discrimination of the VOC profiles between the BR and the GBR (Figure 5). The top 15 discriminants consisted of 5 aldehydes (butanal, (*E*,*E*)-2,4-Decadienal, heptanal, pentanal, and hexanal), 3 alcohols (1-hexanol, 1-Octen-3-ol, and ethanol), 1 ketone (2-heptanone), 2 esters (methyl acetate and ethyl hexadecanoate), and 2-acetyl-1-pyrroline, one of the key compounds contributing to the typical aroma of rice [19]. Then, univariate statistical analyses were further conducted to compare the differences in the amounts of the selected individual VOCs between BR and GBR (Table 1). For the *Japonica* cultivars NJ, LJ, and TH, the amount of myrcene exhibited the most pronounced reduction in GBR as compared with those in BR, ranging from 80.1% to 91.4%. Except for ethyl acetate, ethyl hexadecanoate, and ethanol showing increased levels, most of the listed differential VOCs presented remarkably lower levels in the GBR after germination (Table 1). Similarly, significant decreases in the amounts of the discriminating VOCs were also observed for the *Indica* GBR samples ZZ, ZJZ, and LY (Table 1).

### 3.5. Comparisons between Volatile Profiles of the Japonica and the Indica Subspecies

Owing to the drastic changes in the VOC content induced by the germination process, a PCA was performed for BR and the GBR to compare the overall volatile profiles according to the rice subspecies. The PCA score plot exhibited no clear clustering among BR samples based on the subspecies (Figure S5A), indicating that there were no distinct differences in the volatile compositions between the *Japonica* BR and the *Indica* BR. Moreover, similar results were also found in the PCA score plot for the GBR samples (Figure S5B), accounting for 43.3% of the total variance which were comparable to that in the PCA score plot for BR (46.3%). These findings were also well-supported by the results of the univariate analyses for the single volatiles (Table 1). For example, the contents of the representative aroma compound 2-acetyl-1-pyrroline (2-AP) ranged from 1.59 μg/kg to 1.98 μg/kg in the *Japonica* BR samples NJ, LJ, and TH, which were comparable to those in the *Indica* BR samples ZZ, ZJZ, and LY (1.66–2.25 μg/kg). After the germination treatment, the 2-AP contents of the *Japonica* GBR were decreased to 0.73–0.95 μg/kg, which were still in the same order of magnitude compared with those in the *Indica* GBR (0.51–0.83 μg/kg). Together with similar scenarios observed for the typical VOCs hexanal, heptanal, 1-Octen-3-ol, 1-hexanol, and 2-heptanone, it could be concluded that there were no clear discriminations between the volatile profiles of the *Japonica* and the *Indica* non-fragrant rice, and the composition of the VOCs in the non-fragrant GBR is more likely to be cultivar-dependent rather than subspecies-dependent [19].

## 4. Discussion

In the present study, a broad spectrum of metabolites exhibited increased levels in the GBR compared with those in the BR, independent of rice cultivars and subspecies (Figure 1C). During the germination process, the macromolecular substances such as starch granules and proteins accumulated in seeds were largely degraded into monosaccharides and amino acids, serving as energy sources for sprouting [20]. As a result, a great number of metabolites such as fructose, glucose, alanine, valine, serine, and proline all presented much higher levels in GBR than in BR. Similar notable increases in the levels of sugars and amino acids were also reported in the germination course for wheat [21], barley [22], sorghum [23], and Oat [24]. On the contrary, the amounts of several major free fatty acids in BR, including stearic acid, oleic acid, and linoleic acid, were shown to have decreased in the GBR after germination. For lipid metabolism during germination, the esterified fatty acids are firstly released from the triacylglycerols (TAGs) via lipases catalysis, and the generated free fatty acids (FFAs) are subsequently degraded through *β*-oxidation and glyoxylate cycles, and further converted into sugars for energy and carbon sources [25]. The reduction of lipid contents observed in the present study were also in agreement with the previous reports for wheat [26] and brown rice [27]. On the contrary, the investigations on the germinated oat showed increased levels of lipids [24], which might be attributed to the unchanged or even declined levels of lipase activity during the sprout process [28]. Additionally, it is worth noting that the dynamic changes in the levels of the FFAs are also significantly affected by the sprouting conditions, e.g., temperature, moisture, and germination time [29].

Germination was shown to result in global metabolic changes in rice grains regardless of subspecies. Nevertheless, the different metabolic fingerprints according to subspecies, i.e., the metabolic differences in the BR between the *Japonica* and the *Indica*, were consistently expressed in the GBR after the germination treatment (Figure 2 and Figure 3). A pathway analysis revealed that all the *Japonica* BR showed higher levels of nitrogen-containing metabolites, e.g., serine, alanine, proline, methionine, tryptophan, and glutamic acid, which might be attributed to the variations in the efficiency of nitrogen utilization [30]. *Japonica rice* cultivars are usually distributed across temperate regions, while *Indica* cultivars are mainly cultivated in subtropical and/or tropical zones. This distinction may result in differences in nitrogen assimilation, recycling, translocation, and storage between the *Japonica* and the *Indica* [31], thus leading to the variances in the accumulation of nitrogen-containing metabolites [16]. Interestingly, this subspecies-dependent metabolic fingerprint was also observed in the GBR, especially the essential amino acid tryptophan, methionine, and lysine, which all exhibited more abundance in the *Japonica* GBR than in the *Indica* GBR (Figure 3). In addition, the renowned health-promoting compound γ-aminobutyric acid (GABA), which functions as the predominant inhibitory neurotransmitter as well as being effective in lowering blood pressure [28], was also found to be more accumulated in the *Japonica* GBR. Taking all this into account, the *Japonica* GBR seems to be more nutritious as compared with the *Indica* GBR. 

Rice aroma involves complex sensory attributes with numerous volatile compounds. In the present study, a total of 80 VOCs were identified by means of HS-SPME-GC-MS (Table S2). As the most predominant type of VOCs in BR, aldehydes were thought to provide grassy and floral odor for rice [19]. In this study, the significant decline in the contents of three typical aldehydes, i.e., butanal, pentanal, and hexanal, were observed for all the investigated rice cultivars after germination, with percentage reductions ranging from 42.3% to 85.1% (Table 1). Similarly, the levels of several representative volatiles from the alcohol and ketone classes, inducing 1-hexanol, ethanol, 1-Octen-3-ol, and 2-heptanone, all exhibited drastic decreases in response to the germination process (Table 1). These results were in good agreement with the data from the previous studies by Xia et al. [17] and Wu et al. [18]. Aldehydes, alcohols, and ketones are usually generated from unsaturated fatty acids via oxidation metabolism [19]. In the present study, germination was shown to up-regulate the metabolism of converting lipids into carbohydrates for energy and carbon sources; consequently, less lipids were available for the biotransformation reactions in generating aldehydes, alcohols, and ketones via lipid oxidation and decomposition [17,19].

The aroma volatile 2-acetyl-1-pyrroline (2-AP) was identified as the most prominent compound in the cooked fragrant rice, providing a popcorn-like and/or pandan-like odor [32,33]. Later, 2-AP was also reported to be detected in non-fragrant rice cultivars, but about 6~8 times lower than in aromatic rice [34], and the concentrations were further decreased by 54.2–72.8% for the GBR investigated in the present study (Table 1). Genetic studies on the fragrant trait of the rice lines have revealed that the biosynthesis of 2-AP is controlled by the dominant betaine aldehyde dehydrogenase gene (*Badh2*) located on chromosome 8 [35]. During the germination stage, the elevated expression of *Badh2* was expected to accelerate the process of encoding the active betaine aldehyde dehydrogenase (BADH2) that inhibits 2-AP synthesis by diverting γ-aminobutyraldehyde (GAB-ald), the upstream precursor of 2-AP, into the γ-aminobutyric acid (GABA) [36]. As a result, the level of 2-AP was decreased in the GBR while GABA increased [35,36]. This hypothesis was also well-supported by the data obtained in the present study (Table 1 and Figure 3). These results suggest that the germination treatment may have an adverse impact on the typical flavor profile of the cooked GBR.

## 5. Conclusions

In the present study, a GC-MS-based metabolomics analysis was conducted to investigate the metabolite profiles of the *Japonica* and *Indica* brown rice samples. Despite the pronounced variations in the concentrations of the phytochemicals during the germination process, the subspecies-dependent metabolite signature of the *Japonica* BR, especially the higher levels of essential amino acids and the biofunctional metabolite GABA, were consistently expressed or even amplified in the GBR. By contrast, the typical flavor profile was not improved in response to the germination treatment, and the VOC fingerprints of the GBR were cultivar-dependent rather than subspecies-dependent. Nevertheless, the elaborated data in the present study demonstrated that germination could be utilized as a practical and economic strategy to improve the nutritional quality of brown rice, and that rice from the *Japonica* subspecies seems to be more desirable in terms of the abundance of nutrients for consumers. Further studies are needed to compare the texture and the digestion properties of the GBR in the *Japonica* and the *Indica* subspecies.

## Figures and Tables

**Table 1:** Volatile compounds contributing to the discrimination between brown rice and germinated brown rice samples in the OPLS-DA model.

*Japonica* Cultivars	Brown Rice (BR)															Germinated Brown Rice (GBR)								
Volatile Compounds	NJ				LJ				TH				NJ				LJ				TH			
Myrcene	1.96	±	0.48	b	2.25	±	0.49	b	2.85	±	0.12	a	0.39	±	0.05	c	0.24	±	0.03	c	0.25	±	0.06	c
Butanal	1.34	±	0.06	b	2.28	±	0.40	a	2.03	±	0.29	a	0.32	±	0.05	c	0.36	±	0.04	c	0.35	±	0.04	c
1-Octen-3-ol	3.34	±	0.41	b	4.04	±	0.39	a	4.03	±	0.23	a	1.40	±	0.12	c	1.64	±	0.19	c	1.40	±	0.13	c
Methyl acetate	0.47	±	0.02	c	0.60	±	0.10	c	0.66	±	0.10	c	1.75	±	0.27	b	2.18	±	0.19	a	1.87	±	0.14	b
(*E*,*E*)-2,4-Decadienal	2.70	±	0.31	b	3.51	±	0.29	a	3.32	±	0.29	a	0.91	±	0.19	c	0.93	±	0.28	c	0.82	±	0.14	c
Heptanal	14.38	±	1.93	b	15.82	±	2.25	b	18.32	±	1.50	a	6.33	±	1.39	c	5.75	±	1.25	c	5.98	±	0.55	c
α-Pinene	8.80	±	2.54	a	7.79	±	1.51	a	7.67	±	1.12	a	2.08	±	0.27	b	2.59	±	0.37	b	2.69	±	0.59	b
1-Hexanol	7.07	±	0.58	c	8.21	±	0.39	b	9.46	±	0.48	a	3.13	±	0.83	d	2.97	±	0.70	d	3.17	±	0.51	d
Pentanal	18.37	±	2.78	b	19.03	±	3.29	b	21.95	±	0.74	a	7.62	±	0.86	d	10.55	±	1.02	c	9.32	±	0.72	cd
Hexanal	131.9	±	16.8	c	152.6	±	18.7	b	176.4	±	16.6	a	68.2	±	12.9	d	72.4	±	14.7	d	60.6	±	10.4	d
Ethanol	2.78	±	0.45	c	2.35	±	0.35	c	2.27	±	0.16	c	5.72	±	1.06	a	5.67	±	0.88	a	4.65	±	0.44	b
2-Pentylfuran	2.90	±	0.62	c	3.33	±	0.44	b	4.13	±	0.38	a	0.59	±	0.17	d	0.37	±	0.16	d	0.36	±	0.10	d
Ethyl hexadecanoate	3.35	±	0.41	b	3.91	±	0.34	b	3.52	±	0.58	b	9.53	±	2.13	a	10.67	±	2.53	a	10.59	±	0.87	a
2-Acetyl-1-pyrroline	1.59	±	0.16	b	1.94	±	0.12	a	1.98	±	0.19	a	0.73	±	0.07	d	0.79	±	0.11	d	0.95	±	0.08	c
2-Heptanone	17.05	±	2.20	b	20.96	±	1.19	a	18.91	±	2.36	ab	10.14	±	1.03	c	7.34	±	1.30	cd	7.02	±	1.87	d
*Indica* cultivars	Brown rice (BR)												Germinated brown rice (GBR)											
Volatile compounds	ZZ				ZJZ				LY				ZZ				ZJZ				LY			
Myrcene	2.60	±	0.37	b	2.38	±	0.43	bb	3.34	±	0.66	a	0.22	±	0.02	c	0.42	±	0.04	c	0.27	±	0.06	c
Butanal	2.14	±	0.25	a	1.61	±	0.16		1.93	±	0.25	a	0.32	±	0.01	c	0.32	±	0.04	c	0.36	±	0.04	c
1-Octen-3-ol	4.15	±	0.27	a	3.41	±	0.39	b	4.04	±	0.38	a	1.45	±	0.09	c	1.20	±	0.10	c	1.34	±	0.11	c
Methyl acetate	0.64	±	0.10	d	0.73	±	0.05	d	0.68	±	0.10	d	2.37	±	0.20	a	1.61	±	0.14	c	2.07	±	0.24	b
(*E*,*E*)-2,4-Decadienal	2.86	±	0.29	b	3.53	±	0.13	a	2.53	±	0.33	b	0.92	±	0.10	c	0.89	±	0.23	c	0.76	±	0.18	c
Heptanal	19.31	±	1.77	a	18.44	±	1.33	a	15.54	±	2.56	b	5.36	±	0.88	c	6.54	±	1.14	c	5.78	±	1.01	c
α-Pinene	7.22	±	0.81	a	8.08	±	1.15	a	7.33	±	1.28	a	3.10	±	0.36	b	2.28	±	0.36	b	2.59	±	0.44	b
1-Hexanol	8.03	±	0.37	b	7.41	±	0.43	b	8.69	±	0.91	a	3.65	±	0.35	c	2.87	±	0.49	cd	2.16	±	0.33	d
Pentanal	20.51	±	2.63	b	19.45	±	2.45	b	24.44	±	3.29	a	8.01	±	0.96	c	11.23	±	0.57	c	8.53	±	0.80	c
Hexanal	186.3	±	23.5	a	151.4	±	14.7	b	157.8	±	9.90	b	57.35	±	6.03	c	76.90	±	18.47	c	68.58	±	6.22	c
Ethanol	2.35	±	0.22	d	2.46	±	0.23	d	2.03	±	0.34	d	5.44	±	0.60	c	6.36	±	0.73	b	7.40	±	0.36	a
2-Pentylfuran	4.16	±	0.20	a	3.06	±	0.55	b	1.69	±	0.21	c	0.37	±	0.12	d	0.35	±	0.15	d	0.44	±	0.14	d
Ethyl hexadecanoate	3.92	±	0.43	c	2.89	±	0.30	c	3.34	±	0.41	c	6.86	±	1.00	b	9.75	±	0.98	a	9.96	±	1.36	a
2-Acetyl-1-pyrroline	2.25	±	0.31	a	1.66	±	0.13	b	1.86	±	0.15	b	0.83	±	0.04	c	0.65	±	0.04	cd	0.51	±	0.08	d
2-Heptanone	16.98	±	1.84	b	21.24	±	1.28	a	17.41	±	1.49	b	6.85	±	1.15	d	9.47	±	1.41	c	6.57	±	0.83	d

1. The concentrations of the volatile compounds were expressed as means ± standard deviations in μg/kg equivalents of 1-octanol. 2. Values with different letters (a, b, c or d) in the same row are significantly different (*p* < 0.05).

## Supplementary Materials

The following are available online at https://www.mdpi.com/article/10.3390/foods10102448/s1, Table S1. Metabolites identified by GC-MS in BR and GBR samples. Table S2. VOCs identified by HS-SPME-GC-MS in BR and GBR samples. Figure S1. Exemplary GC chromatogram of the BR. Figure S2. The exemplary interface for the identification of metabolites via MS-DIAL. Figure S3. PCA score plot of the metabolomics data of real samples and the QC sample. Figure S4. The cross-validation parameters and the permutation test result of the OPLS-DA model. Figure S5. PCA score plot of the volatile profiling data.
