# Supplementary material for: Integrated Analysis of Metabolome and Volatile Profiles of Germinated Brown Rice from the Japonica and Indica Subspecies
Source: Foods. 2021 Oct 14;10(10):2448. doi: 10.3390/foods10102448 (PMC8535935; doi:10.3390/foods10102448)
Supplement: Supplementary file 1 [file foods-10-02448-s001.zip › foods-1426930-supplementary.pdf]

foods

Article

Integrated Analysis of Metabolome and Volatile Profiles of Germinated Brown Rice from the *Japonica* and *Indica* Subspecies

Chenguang Zhou [1], Yaojie Zhou [1], Yuqian Hu [1], Bin Li [1], Roujia Zhang [1], Kaiyi Zheng [1], Jie Liu [2], Jing Wang [2], Min Zuo [3] and Siyao Liu [4,*]

[1] School of Food and Biological Engineering, Jiangsu University, Zhenjiang 212013, China; zhouchenguang@ujs.edu.cn (C.Z.); ZhouYaojie2021@gmail.com (Y.Z.); hu13636001423@163.com (Y.H.); lb13753676201@163.com (B.L.); 1000005191@ujs.edu.cn (R.Z.); kaiyizhengjsu@126.com (K.Z.)
[2] China-Canada Joint Lab of Food Nutrition and Health (Beijing), Beijing Technology and Business University, Beijing 100048, China; liu_jie@btbu.edu.cn (J.L.); wangjing@th.btbu.edu.cn (J.W.)
[3] National Engineering Laboratory for Agri-product Quality Traceability, Beijing Technology and Business University, Beijing 100048, China; zuomin1234@163.com
[4] School of Pharmacy, Jiangsu University, Zhenjiang 212013, China
* Correspondence: siyaoliu@ujs.edu.cn

Supplementary Materials

Table S1. Metabolites identified by GC-MS in brown rice and germinated brown rice samples.

compound name	Quant mass	EI similarity	RT [a]	RI [b]
lactic acid	117	966	4.259	1045
glycolic acid	147	935	4.399	1058
alanine	116	873	4.837	1097
hydroxylamine	133	943	5.235	1123
3-hydroxybutyric acid	147	766	5.715	1152
methanolphosphate	241	897	5.796	1158
n-methyl-dl-alanine	130	853	5.943	1167
urea	147	789	6.232	1185
valine	144	989	6.807	1216
4-hydroxybutyric acid	76	901	6.994	1225
hydroxycarbamate	147	751	7.249	1237
pyrophosphoric acid	300	912	7.844	1266
leucine	158	936	7.966	1272
2-aminoethanol	174	987	8.014	1274
isonicotinic acid	180	966	8.041	1275
glycerol	147	972	8.102	1278
norvaline	73	879	8.163	1233
proline	142	896	8.49	1294
succinic acid	147	937	8.572	1301
glycine	174	990	8.796	1310
methylsuccinic acid	147	745	8.915	1315
uracil	241	845	9.17	1326
glyceric acid	147	836	9.296	1331
fumaric acid	245	910	9.459	1338
erythronic acid lactone	147	806	9.871	1371
2,5-dihydroxypyrazine	241	766	9.966	1359
l-serine	204	988	10.071	1363
methylserine	232	880	10.755	1392
threonine	219	984	10.833	1395
β-alanine	174	966	11.748	1431
spermidine	144	871	12.057	1443
homoserine	218	874	12.364	1455
asparagine	100	938	13.085	1483

malic acid	147	900	13.302	1492
salicylic acid	267	752	13.595	1503
l-5-oxoproline	156	928	13.734	1508
l-methionine	176	966	13.911	1515
aspartic acid	232	811	14.149	1524
GABA	174	718	14.452	1535
4-hydroxyproline	140	846	14.564	1540
cysteine	146	844	15.02	2284
2-hydroxyglutaric acid	247	868	15.523	1576
threonic acid	292	933	15.56	1578
dl-2,3-diaminopropionic acid	174	765	16.577	1616
4-hydroxybenzoic acid	267	702	16.655	1620
glutamic acid	246	700	16.744	1621
phenylalanine	142	812	16.757	1623
l-asparagine	116	951	17.961	1670
phthalic acid	147	733	18.165	1678
glycerol-2-phosphate	243	838	19.692	1738
xylonic acid	73	747	22.185	1740
aconitic acid	147	779	19.93	1747
pentonic acid	73	731	22.559	1754
putrescine	174	870	20.247	1657
ribonic acid	73	738	22.729	1761
glutamine	156	955	20.485	1769
3-phosphoglycerate	299	816	20.587	1773
xylonic acid	292	826	21.141	1795
citrulline	157	808	21.944	1828
citric acid	273	960	22.097	1834
cis-aconitic acid	73	828	22.144	1734
glucosamine	73	796	22.614	1928
hexoside	73	752	25.117	1857
asparagine	188	709	23.069	1874
allantoin	331	965	23.549	1894
gulcono-1,4-lactone	217	726	23.889	1909
homocarnosine	154	937	23.944	1911
glucono-1,5-lactone	217	848	24.114	1918
1-methylgalactose	204	756	26.702	1923
4-hydroxycinnamic acid	293	807	24.321	1927
lysine	174	907	24.627	1940
tyrosine	218	962	24.695	1943
galactonic acid	205	724	27.963	1976
pantothenic acid	117	917	25.987	1999
hexitol	318	737	29.048	2023
palmitic acid	117	708	26.906	2040
gluconic acid	147	927	27.021	2045
saccharic acid	147	853	27.572	1996
n-acetyl-d-glucosamine	147	788	27.974	2087
tryptophan	202	893	30.715	2215
serotonin	174	826	35.751	2470
lactulose	204	802	41.54	2648
sucrose	217	939	40.033	2707
trehalose	204	767	42.924	2728
maltose	73	705	43.539	2764
raffinose	217	758	52.558	3354
sitosterol	129	779	52.932	3381
melezitose	133	763	54.344	3484

[a] Retention Time (RT, min) on the DB-5 capillary column.

[b] Retention Index (RI) was auto-generated via MS-DIAL based on the Kovat's index calculation method on a DB-5 capillary column.

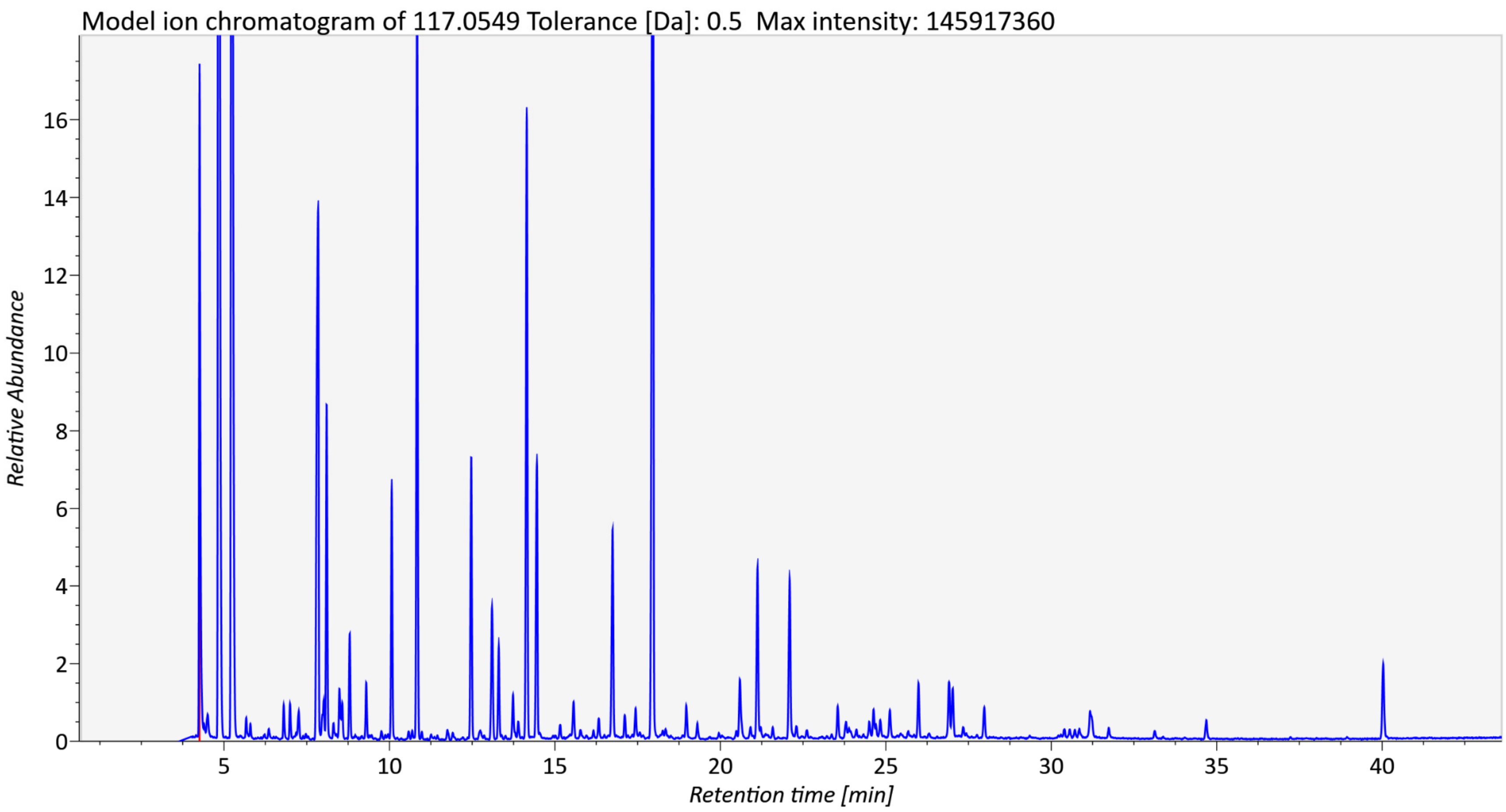

Figure S1. Exemplary GC chromatogram of the brown rice ZJZ from the *indica* cultivar.

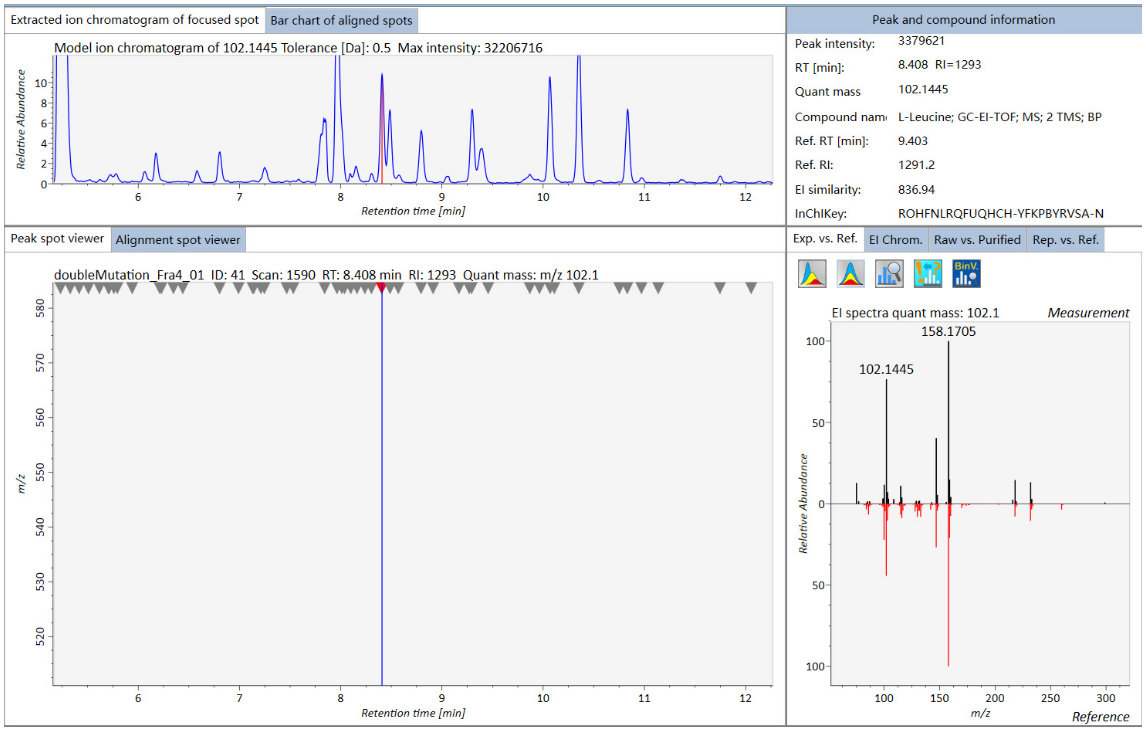

Figure S2. Exemplary interface of the identification of leucine via MS-DIAL.

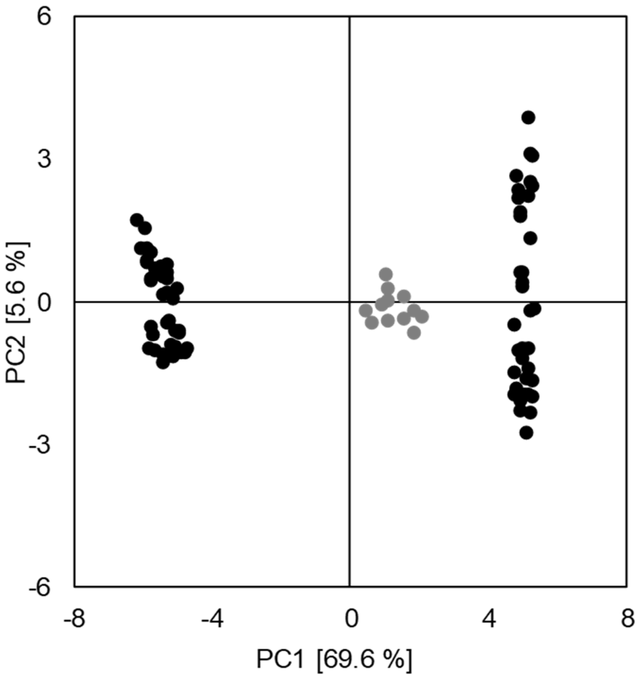

Figure S3. PCA score plot of the metabolomics data of the real samples (black) and the QC sample (gray).

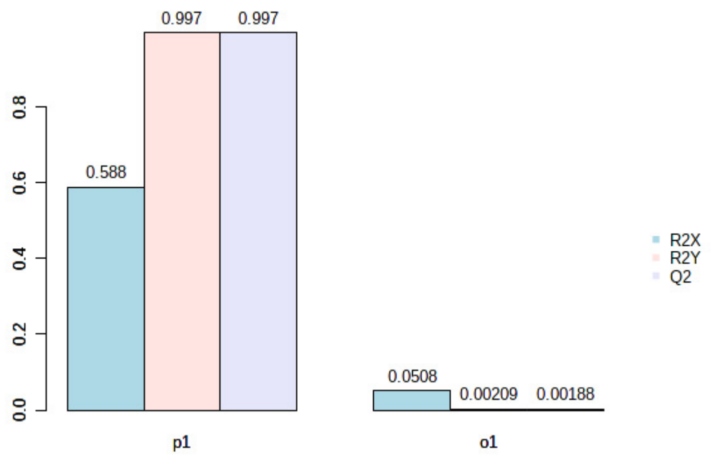

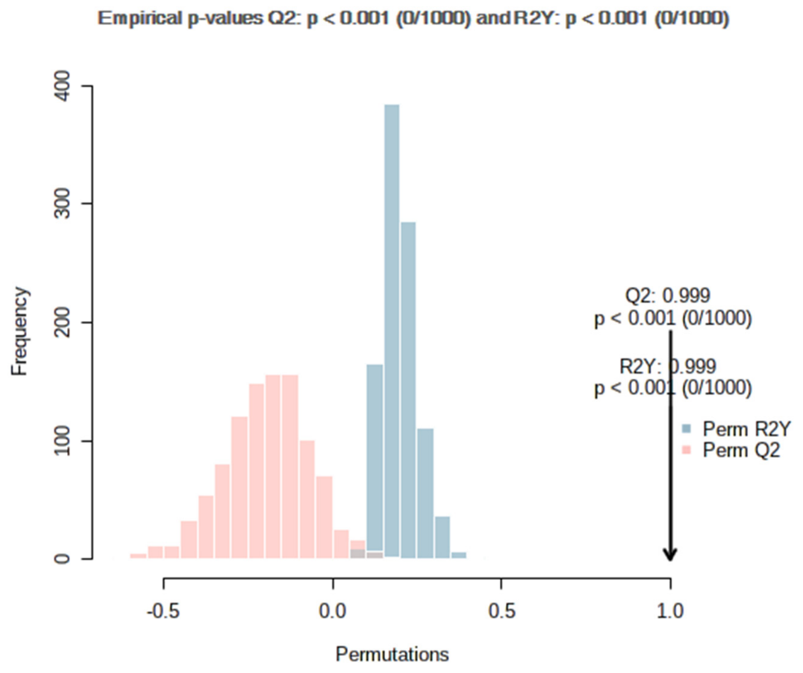

Figure S4. The cross-validation parameters and the permutation test result of the OPLS-DA model.

Table S2. VOCs identified by HS-SPME-GC-MS in brown rice and germinated brown rice samples.

VOCs	m/z	RI ref [a]	RI cal [b]	MS match index
Alkanes				
pentane	43	500	512	940
Heptane	71	700	706	814
Octane	85	800	799	906
Nonane	57	900	897	896
2,2,4,6,6-Pentamethyl-heptane	57	954	953	935
Decane	57	1000	1006	942
Undecane	85	1100	1089	863
Tetradecane	85	1400	1394	873
Alkenes				

1,4-Pentadiene	69	646	660	862
1-Octene	70	842	855	884
1,3-Octadiene	54	954	949	901
Myrcene	93	1145	1149	865
Aldehydes				
2-Methyl-propanal	72	812	828	843
Butanal	44	867	860	829
Methacrolein	70	893	898	959
2-Methyl-butanal	57	907	912	863
3-Methyl-butanal	44	916	920	832
Pentanal	44	975	979	815
Hexanal	56	1079	1076	832
Heptanal	70	1183	1180	858
3-Methyl-2-butenal	84	1215	1219	969
(E)-2-hexenal	98	1220	1225	839
Octanal	84	1293	1288	943
Nonanal	98	1389	1384	851
(E)-2-Octenal	70	1420	1422	808
Decanal	82	1484	1481	948
(E,E)-2,4-Heptadienal	81	1498	1495	951
Benzaldehyde	105	1530	1520	914
(E)-2-nonenal	70	1532	1530	817
(E,E)-2,4-Nonadienal	81	1699	1705	896
(E)-2-Undecenal	121	1750	1755	879
(E,E)-2,4-Decadienal	81	1798	1802	913
3-Methyl-hexanal	70	nf	1130	845
3,3-Dimethyl-hexanal	84	nf	1206	866
Furans				
Furan	68	797	799	929
2-Methylfuran	82	817	822	922
Tetrahydrofuran	42	829	835	907
3-Methylfuran	82	858	849	821
2-Ethylfuran	81	960	955	893
2-Propylfuran	81	1011	1015	968
2-Butyl furan	81	1123	1130	807
2-Pentylfuran	83	1231	1237	773
2-Acetyl-5-methylfuran	109	1593	1599	916
Ketones				
2-Butanone	43	893	890	892
2,3-Butanedione	43	970	971	920
2-Methyl-3-pentanone	57	997	1000	782
3-Penten-2-one	84	1132	1139	864
2-Heptanone	58	1180	1175	824
6-Methyl-2-heptanone	58	1237	1230	827
2-Hydroxy-3-butanone	45	1280	1284	816
2-Octanone	43	1297	1290	894
2-Nonanone	58	1387	1392	846
3-Octen-2-one	55	1414	1420	812
3,4,5-Trimethyl-2-cyclopenten-1-one	109	1483	1490	828
4,4-Dimethyl-2-cyclopenten-1-one	67	1511	1505	856
Alcohols				
Ethanol	46	939	935	853
1-Pentanol	56	1252	1259	901
1-Hexanol	57	1360	1366	954
Cyclohexanol	57	1393	1399	957
1-Heptanol	57	1444	1450	905
1-Octen-3-ol	70	1453	1456	937
2-Ethylhexanol	56	1480	1470	877

n-Octanol	93	1558	1562	894
Benzyl alcohol	42	1865	1870	939
2-Phenylethanol	91	1912	1920	861
Esters				
Methyl acetate	74	825	827	842
ethyl acetate	43	894	900	956
Ethyl hexadecanoate	101	2241	2250	803
ethyl oleate	97	2470	2460	933
Caproic acid vinyl ester	99	nf	1300	881
Others				
α-Pinene	107	1026	1030	902
2-Acetyl-1-pyrroline	94	1317	1322	937
Acetic acid	60	1479	1485	888
Hexanoic acid	60	1849	1840	827
Heptanoic acid	60	1957	1950	884
Phenol	107	2008	2009	821
Nonanoic acid	60	2174	2170	932
4-Ethylphenol	81	2183	2180	895
Decanoic acid	73	2279	2275	955
Indole	107	2448	2450	946

[a] RI ref, the Kovats' retention index information obtained from the NIST Chemistry WebBook database (https://webbook.nist.gov/chemistry/name-ser/).

[b] RI cal, the experimental Kovat's retention index calculated based on a DB-WAX capillary column.

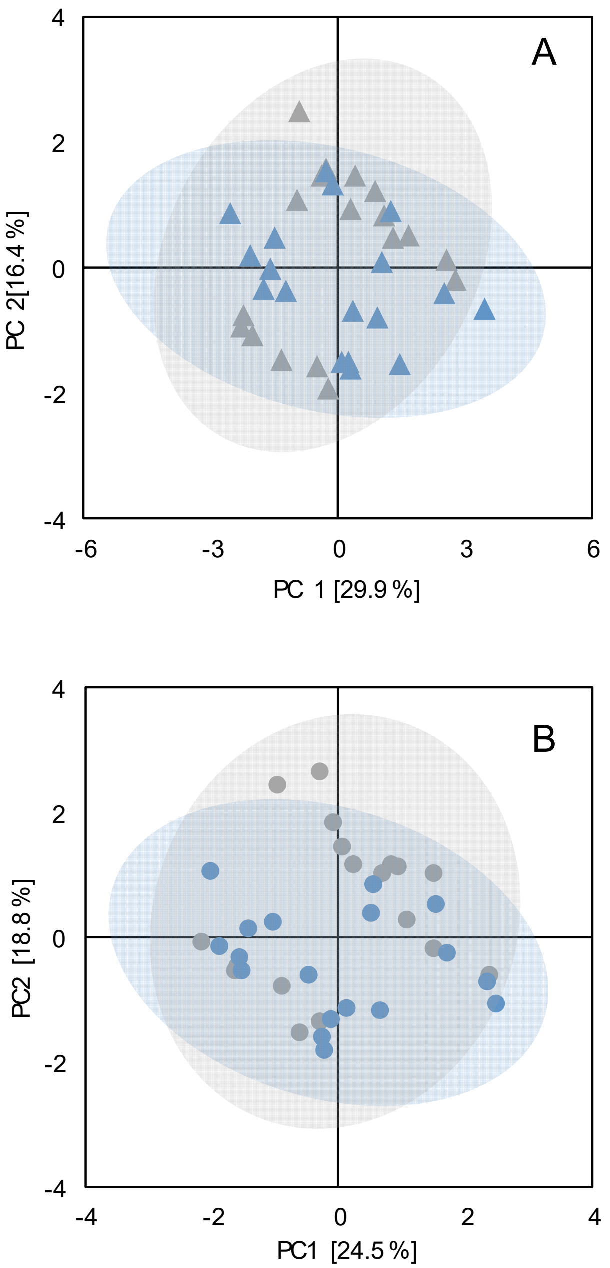

Figure S5. PCA score plot of the volatile profiling data of the brown rice (A) and the germinated brown rice (B) from the *indica* cultivars (triangle) and the *japonica* cultivars (circle); the boundaries of the clusters correspond to the 95% Hotelling's T2 ellipses. .
